# Supplementary material for: Long-term clinical and safety outcomes from a single-site phase 1 study of neural stem cell transplantation for chronic thoracic spinal cord injury
Source: Cell Rep Med. 2024 Dec 2;5(12):101841. doi: 10.1016/j.xcrm.2024.101841 (PMC11722094; doi:10.1016/j.xcrm.2024.101841)
Supplement: Document S1. Table S1 [file mmc1.pdf]

Cell Reports Medicine, Volume *5*

Supplemental information

Long-term clinical and safety outcomes from a single-site phase 1 study of neural stem cell transplantation for chronic thoracic spinal cord injury

Joel R. Martin, Daniel Cleary, Mickey E. Abraham, Michelle Mendoza, Betty Cabrera, Catriona Jamieson, Martin Marsala, and Joseph D. Ciacci

SUPPLEMENTARY MATERIAL

Table S1: Supplementary electrophysiology summary data. Related to Table 2, Figure 5.

<table>
<tr><td>Subject 001</td><td>

Week 4

some activity T6-10 RA and PS bilaterally
none in right Rectus femoris

Week 6

some activity T7-9 RA and PS bilaterally
none in right Rectus femoris

Month 6

some activity T7-9 RA and PS bilaterally
none in right Rectus femoris

Month 12

some activity T7-9 RA and PS bilaterally
none in right Rectus femoris

Month 18

only BMCA done.

Month 27

PSL:

Multifidus: T9 with many MUPs; T10 with none
Superficial: T10 with many MUPs; T11 with none/+spont

PSR:

Multifidus: T10 with several MUPs; T11 with none/+spont
Superficial: T10 with several MUPs; T11 with none/+spont

RAL: Upper segment with many MUPs
RAR: Upper segment with several MUPs
LL bilaterally: no voluntary MUPs; +spont LVm, RTA, RVm
BMCA: newly developed muscle responsiveness in lower limbs to reinforcement maneuvers

Month 36

No EMG changes.

Month 60

EMG: Needle EMG now indicates some voluntary control of rectus abdominis bilaterally at T11 and T12 at the left, not seen previously. No MUPs under voluntary control at T11 on the right, as before.
BMCA: Constant EMG activity was recorded from the right tibialis anterior muscle and constant, low amplitude movement of the right great toe was seen. This seemed to be involuntary (spontaneous).

</td></tr>
<tr><td>Subject 006</td><td>

Week 12

PSL or MTF: no MUPs T7 or below
PSR or MTF: MUPs noted at T6-8
RAR: no MUPs at T7 (presumably lower-mid segment)

Month 12

PSL

Superficial: MUPs noted at T6-8
Multifidus: T6 with many MUPs; T7 with none

PSR

Superficial: MUPs noted at T6-8
Multifidus: T6 with many MUPs; T7 with none

RAL: no MUPs
RAR: 2 MUPs distant in upper segment

Month 18

PSR: new intolerance to study at T9 and above
PSL: new intolerance to study at T9 and above

T9 and 10 with 1-2 superficial MUPs (not tested before)

RA: new intolerance to study at T9 and above

no definite MUPs at T9-T10

Month 36

Continued intolerance to study at T9 and above. Examined T12,T11,T10 bilaterally
RAR

</td></tr>
</table>

	▪ Right rectus abdominus at T10- +2 spont activity, +2 complex repetitive discharges, 1 voluntary MUP at T11- no spont activity/discharges, no MUPs at T12- +1 spont activity, no complex repetitive discharges, 2 voluntary MUPs<br>o RAL<br>▪ At T10 +1spont activity, +2complex repetitive discharges, no voluntary MUPs<br>▪ At T11- no spont activity/discharges, no MUPs<br>▪ At T12- no spont activity/discharges, 1 MUP (possibly 2) under voluntary control<br>o Control of rectus abdominus muscles bilat at T10 and to a lesser extent at T12 ; At T10, level of activation seemed better on left;<br>o EMG findings were not present in the T10-T12 paraspinal muscles<br>• Month 42<br>o EMG: right sural sensory amplitude is larger than last study, but still significantly smaller than left. Right tibial motor CMAP lower limb EMG is unchanged. Rectus abdominus at and below umbilicus (T10-T12) shows no voluntary MUPs. MEPs still do not show continuity of central motor pathways to lower trunk and lower limbs.<br>• Month 48<br>o EMG: Sural sensory potential is now present and quite normal (suggesting technical problem during last study), right rectus abdominis did not show any MUPs. rectus abdominus at T11 with 1 MUP and T12 with 2-3 MUPs; Paraspinal muscles showed MUPs under voluntary control at T10-T11 bilaterally, but not at T12.<br>o BMCA: no change<br>• Month 54<br>o EMG No change<br>o BMCA: No change from baseline, touch of the patient introduced 60 Hz interference<br>• Month 60<br>o EMG: left superficial peroneal sensory response is absent now. Rectus abdominis EMG did not show MUPs on either side. T11-T12 Paraspinals not studied due to T8-T10 showing no MUPs during visit. Lower Limb EMG showed no voluntary MUPs<br>o BMCA: suggestion of response of right tibialis anterior to reinforcement maneuver
Subject 008	• Month 12<br>o RAR: no activation, unclear sampling<br>• Month 18<br>o RA: no activation bilaterally<br>o PSR: need to confirm level of landmark (8cm above base of scar)<br>o PSL: need to confirm level of landmark (13cm above base of scar)
Subject 010	• Week 12<br>o RAL: 2-3 hard to activate MUPs likely in superior segment<br>o RAR: no MUPs<br>o PSL (Not tested above T9 due to scar)<br>▪ Multifidus: None at T9 or below<br>▪ Superficial: None at T9 or below<br>o PSR<br>▪ Multifidus: T6 with many MUPs, T7 with none, +spont<br>▪ Superficial: not reported as different to Multifidus.<br>• Month 6<br>o RAL: No MUPs (likely lower segment tested)<br>o RAR: not tested<br>o PSL: Voluntary MUPs in left T5-T8 (likely superficial)<br>o PSR: 1 MUP in T7 (unclear if superficial or Multifidus); T8 with none<br>• Month 18<br>o LL bilaterally: No MUPs, mild spontaneous activity in right TA<br>o PSL<br>▪ Multifidus: T6 with chronic neurogenic changes; T7 with none<br>▪ Superficial: T8 with several units; T9 with none<br>o PSR<br>▪ Multifidus: T6 with 2-3 voluntary units; T7 none<br>▪ Superficial: T7 with 1-2 MUPs; T8 with none

	◦ RAL: 2-3 hard to activate MUPs likely in superior segment<br>◦ RAR: no MUPs<br>• Month 36<br>◦ EMG: SPSR reconfirmed at T7<br>◦ BMCA: Deep tendon reflexes increased at knees<br>• Month 42<br>◦ EMG: No motor units under voluntary control in lower limbs<br>◦ BMCA: Deep tendon reflexes increased at knees, suggestion of response of right medial hamstring to reinforcement maneuver

EMG electromyography, BMCA brain motor control assessment, PS paraspinal, PSL paraspinal left, PSR paraspinal right, SPSR superficial paraspinal right, RA rectus abdominis, RAL rectus abdominis left, RAR rectus abdominis right, LL lower limbs, LVm left vastus medius, RTA right tibialis anterior, RVm right vastus medius, MTF multifidus, MUP multi-unit potential, CMAP, MEP motor evoked potential.
