# Supplementary material for: Long-term clinical and safety outcomes from a single-site phase 1 study of neural stem cell transplantation for chronic thoracic spinal cord injury
Source: Cell Rep Med. 2024 Dec 2;5(12):101841. doi: 10.1016/j.xcrm.2024.101841 (PMC11722094; doi:10.1016/j.xcrm.2024.101841)
Supplement: Document S2. Article plus supplemental information [file mmc2.pdf]

Cell Reports
Medicine

Article

Long-term clinical and safety outcomes from a single-site phase 1 study of neural stem cell transplantation for chronic thoracic spinal cord injury

Graphical abstract

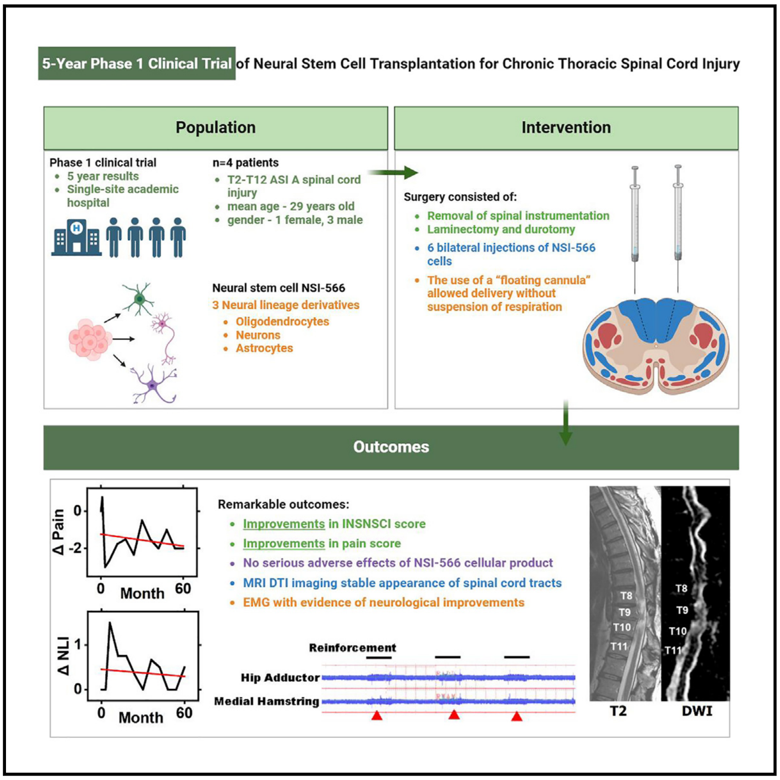

Highlights

- NSI-566 transplanted in the spinal injury site of patients can be performed safely
- NSI-566 transplantation was shown to decrease post-operative pain
- NSI-566 transplantation was shown to improve motor and sensory function
- NSI-566 transplantation resulted in electrophysiological defined improvement

Authors

Joel R. Martin, Daniel Cleary, Mickey E. Abraham, ..., Catriona Jamieson, Martin Marsala, Joseph D. Ciacci

Correspondence

jciacci@ucsd.edu

In brief

Pre-clinical studies of spinal cord-derived neural stem cell (NSI-566) for spinal cord injury showed promising results. Martin et al. report the results of the 60-month study of a phase 1 first-in-human clinical trial of perilesional NSI-566 implantation in four patients with chronic ASIA-A thoracic spinal cord injury.

Check for updates

Martin et al., 2024, Cell Reports Medicine *5*, 101841
December 17, 2024 Published by Elsevier Inc.
https://doi.org/10.1016/j.xcrm.2024.101841

CellPress

Cell Reports Medicine

CellPress
OPEN ACCESS

Article

Long-term clinical and safety outcomes from a single-site phase 1 study of neural stem cell transplantation for chronic thoracic spinal cord injury

Joel R. Martin,[1] Daniel Cleary,[1] Mickey E. Abraham,[1] Michelle Mendoza,[2] Betty Cabrera,[2] Catriona Jamieson,[2] Martin Marsala,[3] and Joseph D. Ciacci[1,4,*]

[1]Department of Neurological Surgery, University of California, San Diego, La Jolla, CA 92037, USA

[2]Department of Medicine, Division of Regenerative Medicine and CIRM Alpha Stem Cell Clinic, University of California, San Diego, La Jolla, CA 92037, USA

[3]Department of Anesthesiology, University of California, San Diego, La Jolla, CA 92037, USA

[4]Lead contact

*Correspondence: jciacci@ucsd.edu

https://doi.org/10.1016/j.xcrm.2024.101841

SUMMARY

We report the long-term results for a phase 1 study of neural stem cell transplantation for chronic spinal cord injury. The trial was registered on ClinicalTrials.gov as NCT01772810. The primary outcome of the trial was to test the feasibility and safety of human spinal cord-derived neural stem cell (NSI-566) transplantation for the treatment of chronic spinal cord injury in four subjects with thoracic two to thoracic twelve spinal cord injury. Here, we report that all four subjects tolerated the stem cell implantation procedure well, and two subjects had durable electromyography-quantifiable evidence of neurological improvement as well as increased neurological motor and sensory scores at five years post-transplantation.

INTRODUCTION

Spinal cord injury (SCI) is a devastating condition that results in significant impairment of millions of individuals worldwide.[1] Management of SCI primarily focuses on stabilizing the injury, preventing further damage, and rehabilitating the patient to potential functional recovery.[2,3] Traditional treatments include surgery and neurological rehabilitation. In recent years, neuromodulation[3] and cell-based therapies[4] have emerged as promising procedures for SCI. Among the various types of stem cells, fetal-derived neural stem cells (NSCs) have a favorable safety profile due to their established lineage commitment potential and lack of teratoma formation.

Previously, we reported on the safety and tolerability of implantation of the NSC line NSI-566 in chronic thoracic complete SCI patients.[5] The NSI-566 line is a human NSC line authorized by the Food and Drug Administration (FDA) for clinical testing.[6–10] Since the initial safety report[5] that followed patients for 18 months in a 60-months study, several human trials using NSC for SCI have described similar results.[11–13] Here we report the long-term extension of the previously documented 18-months outcome results of the first cohort of patients enrolled in a five-year phase 1 first-in-human clinical trial of implantation of an NSC product, NSI-566, into the injury site of patients with chronic ASI-A grade thoracic SCI.

RESULTS

Phase 1 clinical study design

Inclusion and exclusion criteria are previously described in our initial report.[5] The patient's surgery was at least one year but no more than two years after traumatic SCI, classified as AISA-A, and with levels T2–T12. No control group was included (Figure 1). All subjects received spinal cord injections of human spinal cord-derived NSCs (NSI-566). The trial was registered on ClinicalTrials.gov as NCT 01772810. IRB approval was granted by UCSD Health Center, Human Research Protections Program (HRPP), 9452 Medical Center Drive, La Jolla, CA 92037. A total of four subjects received NSI-566 spinal cord implantation with a post-procedure follow-up of five years. All subjects tolerated the procedure well with no serious adverse events (SAEs) in the immediate post-procedure period. Prospective data were collected including International Standards for the Neurological Classification of Spinal Cord Injury (ISNCSCI) scores, functional and pain surveys, Spinal Cord Independence Measure (SCIM) scores, electromyography (EMG), Brain Motor Control Assessment (BMCA), and serial MRI. The presence of donor-specific human leukocyte antigen (HLA) antibodies was also monitored periodically.

NSI-566

NSI-566 was derived from a single postmortem spinal cord of an eight-week gestational age fetus and was obtained in compliance with the National Institutes of Health (NIH) and FDA Good Tissue Practice Guidelines. NSI-566 was provided as a live-cell suspension and was prepared one day prior to each scheduled surgery at a cGMP facility. One or more vials of the cryopreserved CCB were thawed at once, washed of the freezing medium by repeated centrifugation in a hibernation medium (HM), and concentrated to a final concentration of 2×10^6 cells/mL of HM. This target concentration had been established for being

This is an open access article under the CC BY license (http://creativecommons.org/licenses/by/4.0/).

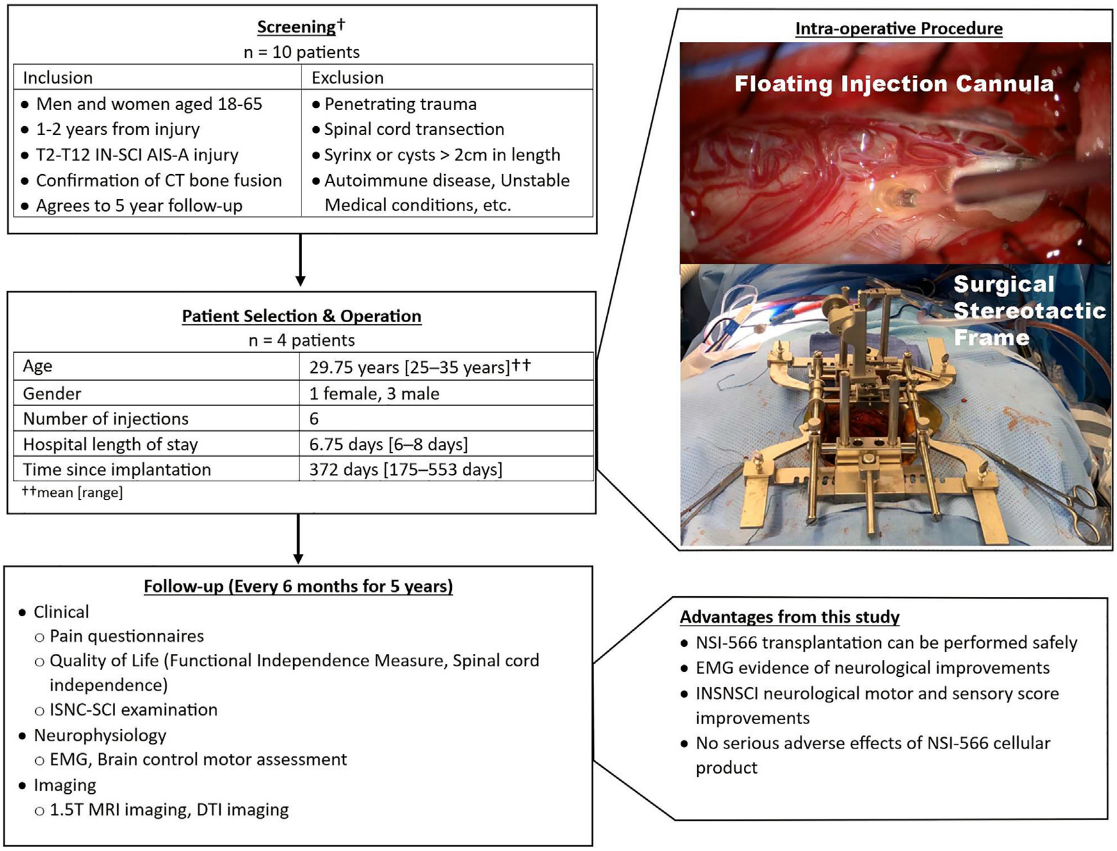

Figure 1. Eligibility screening, patient selection, intra-operative procedure, follow-up, and advantages noted from this study
†See Curtis et al. for complete screening criteria.

safe and adequate for intraspinal injections by series of pre-clinical[7,9] and clinical studies.[10]

Neural stem cell implantation

The NSI-566 injections were delivered at a dose of 2×10^5 cells per injection site bilaterally into the remaining tissue lateral to the injury site with the aid of a floating cannula and within the medial white matter-appearing tracts of approximately one segment below the injury site, as verified by intra-operative fluoroscopy imaging. Injections were made using a customized stereotactic cell injection device.[14]

Adverse events

Adverse events are reported in Table 1. There were 65 adverse events recorded, but only one SAE in the four transplanted patients (0.25 SAEs per patient). Subject 008 died from complications from sepsis related to a sacral ulcer after 30 months post-transplant.

Neurological responses

ISNCSCI exams were performed at initial screening, four weeks, and 12 weeks in the post-surgical period, and then at every six months until study completion (Table 2). The most rostral injection site was the first segment caudal to the neurological level defined by the initial ISNCSCI examination. Two patients (001 and 010) experienced improvements in neurological level of injury (NLI), motor score, and sensory score as demonstrated by physical exam (Figure 2). Compared to neurological score at two years after cell grafting,[5] subject 001 experienced a decline from two levels of improvement (at two years) to one level of improvement at five years. Subject 010 improvement remained stable at one level of neurological improvement at both two and five years.

Withdrawal of immunosuppression

In all four subjects, tacrolimus and mycophenolate mofetil were withdrawn at 12 weeks post-transplantation. Subject 010 developed positive anti-HLA antibodies at six months. Further analysis revealed that the measured anti-HLA antibodies of CW1, CW8, DRB1*04:04, and DR16 were not antibodies with specificity against the HLA alleles of the donor cells. The subject denied additional transfusions or blood products post-implantation, and a bystander immune response was ruled out when months 12 and 18 revealed similar anti-HLA antibody results. It was concluded that the immunoreactivity present in the patient was not related to the NSC treatment.

Pain and spinal cord independence measurements

Two of the four patients (001 and 006) had overall decreased pain scores post-operatively (Figure 3), which included both

Table 1. Summary of adverse events in all 4 transplanted patients

Adverse event	Total	Serious	Relationship to study drug	Relationship to immuno-suppressant	Relationship to surgery	Relationship to surgical device
Cardiac disorders	0	0	0	0	0	0
Gastrointestinal disorders	18	0	0	0	0	0
General disorders and administration site conditions	8	0	0	0	0	0
Infections and infestations	9	1	0	0	0	0
Injury, poisoning, and procedural complications	9	0	0	0	3	0
Investigations	0	0	0	0	0	0
Metabolism and nutrition disorders	2	0	0	0	0	0
Musculoskeletal and connective tissue disorders	0	0	0	0	0	0
Nervous system disorders	4	0	0	0	0	0
Psychiatric disorders	4	0	0	0	0	0
Renal and urinary disorders	1	0	0	0	0	0
Reproductive system and breast disorders	0	0	0	0	0	0
Respiratory, thoracic, and mediastinal disorders	2	0	0	0	0	0
Skin and subcutaneous tissue disorders	8	0	0	0	0	0
Vascular disorders	0	0	0	0	0	0
Total	65	1	0	0	3	0

discomfort and allodynia in the transition zone, surgical site pain, and neuropathic and nociceptive pain elsewhere. Patient 008 experienced mildly increased pain, and patient 010 pain scores remained mostly constant. Quality of life SCIM scores for three patients (001, 006, and 010) saw a nonsignificant change. Patient 008 experienced a large decline in SCIM score due to an SAE at 30 months due to a sacral ulcer.

Dynamic response assessment by MRI and DTI imaging

MR imaging for all patients demonstrated varying degrees of focal spinal cord myelomalacia (Figure 4). There was no radiographical evidence of immediate or delayed complications after the NSC injections, including no new areas of cord or soft-tissue edema, enhancement, or development of swelling or fluid collections on immediate post-procedural or follow-up imaging. No visible morphologic change was observed in the spinal cord myelomalacia on either the pure anatomic or diffusion tensor sequences. In all four patients, diffuse tensor imaging (DTI) imaging revealed a stable appearance of spinal cord tracts both at the injury site and rostral/caudal to the injury site but did not show extensive evidence of remodeling or improvement of tractography.

Neurophysiologic responses

In subject 001 (T8 level of injury), EMG showed activity at left T9 and right T10 rectus abdominis and paraspinal muscles at four weeks post-transplant (Table S1). This improved to more prominent bilateral T10 paraspinal activity at 27 months. BMCA also showed newly developed muscle responsiveness in lower limbs to reinforcement maneuvers at 27 months. At 60 months, constant EMG activity was recorded from the right tibialis anterior muscle and right toe, and needle EMG also indicated some new voluntary control of rectus abdominis bilaterally at T11 and T12 at the left. In subject 006 (T7 level of injury), 12-month EMG analysis showed new voluntary activity in the right rectus abdominus and bilateral T6 to T8 paraspinal muscles. At 18 months post-grafting, the patient developed sensation during EMG needle insertion at T9 bilaterally. From 36 to 54 months, new EMG analysis showed improved control of rectus abdominus muscles bilaterally at T10 and paraspinal muscles at T10–T11. At 60 months, BMCA showed newly developed muscle responsiveness in lower limbs to reinforcement maneuvers (Figure 5). Subject 010 (T5 level of injury) continued to show EMG activity in the right superficial paraspinal muscle at T7 from six to 36 months post-transplant, and BMCA showed suggestion of newly developed muscle responsiveness in lower limbs to reinforcement maneuvers at 42 months.

DISCUSSION

Embryonic stem cells were among the first reservoirs of pluripotent stem cells to be utilized for use in SCI cell-based therapy research.[5,15,16] Recently, Levi and colleagues conducted a phase 2 trial using a fetal brain-derived human CNS stem cell line (HuCNS-SC) in chronic SCI patients with cervical injury.[11] In the study, six patients were transplanted with varying cell doses to define an optimal dose. This derived optimal dose was applied to a treatment group of six new patients and

Table 2. Timeline of post-operative outcome measures

		Baseline	Wk 4	Wk 12	Mo 6	Mo 12	Mo 18	Mo 27	Mo 30	Mo 36	Mo 42	Mo 48	Mo 54	Mo 60
Subject 001<br>29 yo<br>T8 NLI	NLI SR	T8	T8	T8	T10	T10	T10	T9	UTT	T9	UTT	UTT	UTT	T9
	NLI SL	T8	T8	T8	T10	T10	T10	T9	–	T9	–	–	–	T9
	NLI MR	T8	T8	T8	T10	T10	T10	T9	–	T9	–	–	–	T9
	NLI ML	T8	T8	T8	T10	T10	T10	T9	–	T9	–	–	–	T9
	NLI	T8	T8	T8	T10	T10	T10	T9	–	T9	–	–	–	T9
	HLA	Neg	ND	Neg	ND	Neg	Neg	Neg	–	ND	–	–	–	ND
	FIM	109/128	87/128	109/128	109/128	ND	ND	ND	–	ND	–	–	–	ND
	SCIM	67/100	64/100	67/100	67/100	67/100	67/100	67/100	–	69/100	–	–	–	67/100
	Pain	6/10	8/10	0/10	0/10	0/10	2/10	0/10	–	ND	–	–	–	4/10
Subject 006<br>35 yo<br>T7 NLI	NLI SR	T7	T7	T7	ND	T7	T7	T7	T7	T7	T7	T7	T7	T7
	NLI SL	T8	T7	T7	ND	T7	T7	T7	T7	T7	T7	T7	T7	T7
	NLI MR	T7	T7	T7	ND	T7	T7	T7	T7	T7	T7	T7	T7	T7
	NLI ML	T8	T7	T7	ND	T7	T7	T7	T7	T7	T7	T7	T7	T7
	NLI	T7	T7	T7	ND	T7	T7	T7	T7	T7	T7	T7	T7	T7
	HLA	Neg	ND	ND	ND	Neg	Neg	ND	ND	ND	ND	ND	ND	ND
	FIM	109/128	ND	109/128	109/128	ND	ND	ND	ND	ND	ND	ND	ND	ND
	SCIM	74/100	74/100	74/100	74/100	74/100	74/100	74/100	72/100	72/100	72/100	70/100	70/100	70/100
	Pain	6/10	2/10	3/10	4/10	3/10	3/10	2/10	2/10	3/10	2/10	5/10	4/10	4/10
Subject 008<br>37 yo<br>T2 NLI	NLI SR	T2	T2	–	–	T2	T2	T2	T2	–	–	–	–	–
	NLI SL	T2	T2	–	–	T2	T2	T2	T2	–	–	–	–	–
	NLI MR	T2	T2	–	–	T2	T2	T2	T2	–	–	–	–	–
	NLI ML	T2	T2	–	–	T2	T2	T2	T2	–	–	–	–	–
	NLI	T2	T2	–	–	T2	T2	T2	T2	–	–	–	–	–
	HLA	ND	PRA I neg<br>PRA II: "Weakly positive"	–	–	Neg	Neg	ND	ND	–	–	–	–	–
	FIM	109/128	82/128	–	–	ND	ND	ND	ND	–	–	–	–	–
	SCIM	67/100	67/100	–	–	67/100	67/100	67/100	45/100	–	–	–	–	–
	Pain	4/10	6/10	–	–	6/10	5/10	7/10	7/10	–	–	–	–	–

(*Continued on next page*)

Table 2. *Continued*

		Baseline	Wk 4	Wk 12	Mo 6	Mo 12	Mo 18	Mo 27	Mo 30	Mo 36	Mo 42	Mo 48	Mo 54	Mo 60
Subject 010	NLI SR	T5	T5	ND	T6	T6	T6	UTT	UTT	T6	T6	UTT	UTT	UTT
27 yo	NLI SL	T5	T5	ND	T7	T7	T7	–	–	T7	T7	–	–	–
T5 NLI	NLI MR	T5	T5	ND	T6	T6	T6	–	–	T6	T6	–	–	–
	NLI ML	T5	T5	ND	T7	T7	T7	–	–	T7	T7	–	–	–
	NLI	T5	T5	ND	T6	T6	T6	–	–	T6	T6	–	–	–
	HLA	Neg	ND	ND	positive for HLA I and II	positive for HLA I and II	positive for HLA I and II	–	–	ND	ND	–	–	–
	FIM	109/128	90/128	109/128	109/128	ND	ND	–	–	ND	ND	–	–	–
	SCIM	75/100	75/100	75/100	75/100	75/100	75/100	–	–	75/100	73/100	–	–	–
	Pain	0/10	3/10	0/10	0/10	0/10	0/10	–	–	0/10	0/10	–	–	–

yo, years old; NLI, neurological level of injury; SR, sensory right; SL, sensory left; MR, motor right; ML, motor left; FIM, functional independence measure; SCIM, spinal cord independence measure; ND, not done; UTT, unable to travel.

compared to a control group. Transplantation with HuCNS-SC was deemed safe, feasible, and well tolerated in the study. Trends toward improvement in motor function and spasticity were also observed. Similarly, our human NSC study proved to be safe.

As described, there were 65 adverse events recorded, but only one SAE. Subject 008 died from complications from sepsis that was likely related to a sacral ulcer after 30 months post-transplant. This serious event was most likely not directly attributed to NSI-566 or from surgery. Both immunosuppression drugs, tacrolimus and mycophenolate mofetil, are withdrawn at 12 weeks post-transplantation. From examination reports, subject 008 did not have a sacral ulcer at the beginning of the study. Sacral ulcers can be common in patients with neurological injury,[17] but immunosuppression cannot be ruled out as a potential contributor to the cause of the sacral ulcer and ultimate sepsis infection. The family of subject 008 did not wish to perform an autopsy.

It is unclear when NSCs should be transplanted after the initial SCI. Our study focused on chronic SCI of at least greater than one year after injury.[5,11,18,19] Data from pre-clinical studies suggest that transplantation in the subacute period can contribute to improved remyelination of axons in SCI, as compared to several months after injury.[20] Likewise, a few studies have looked at implantation of stem cells in the immediate injury period. Bone-marrow-derived mesenchymal stromal cells (MSCs) have been shown to migrate to the site of injury, and more favorable effects have been observed when transplantation of MSCs occurred a week after, rather than immediately after injury,[21,22] but it did not have durable effects. The role of immunity likely has a role in this observed delayed effect. Cumulative data suggest that the pro-inflammatory microenvironment following SCI results in suboptimal conditions for NSC regeneration and differentiation thereby necessitating an immunosuppressive regimen.[11,23] In this study, tacrolimus and mycophenolate mofetil were used with no serious adverse effects due to medications.

Imaging studies also showed no concern for adverse effects, including no tumor growth, or concern for infection or immune rejection. As described in our preliminary paper, DTI was performed for research purposes. DTI enables both qualitative and quantitative assessment of the spinal cord and could reveal functional tracts of the spinal cord in SCI that could be improved after stem cell implantation.[24,25] In our study, DTI revealed a stable appearance of spinal cord tracts both at the injury site and rostral/caudal to the injury site but did not show extensive evidence of remodeling or improvement of tractography.

Secondary measures included pain, ISNCSCI exam, and electrophysiological tests. Two of the four patients had overall decreased pain scores post-operatively, which included both discomfort and allodynia in the transition zone, surgical site pain, and neuropathic and nociceptive pain elsewhere. ISNCSCI exams were performed until study completion. At the end of the study (42–60 months post-transplant), two patients experienced improvements in NLI, motor score, and sensory score. Likewise, BMCA and EMG revealed new activity in lower-extremity muscle groups. EMG also revealed voluntary control of the rectus abdominis muscle below the level of injury in two subjects and

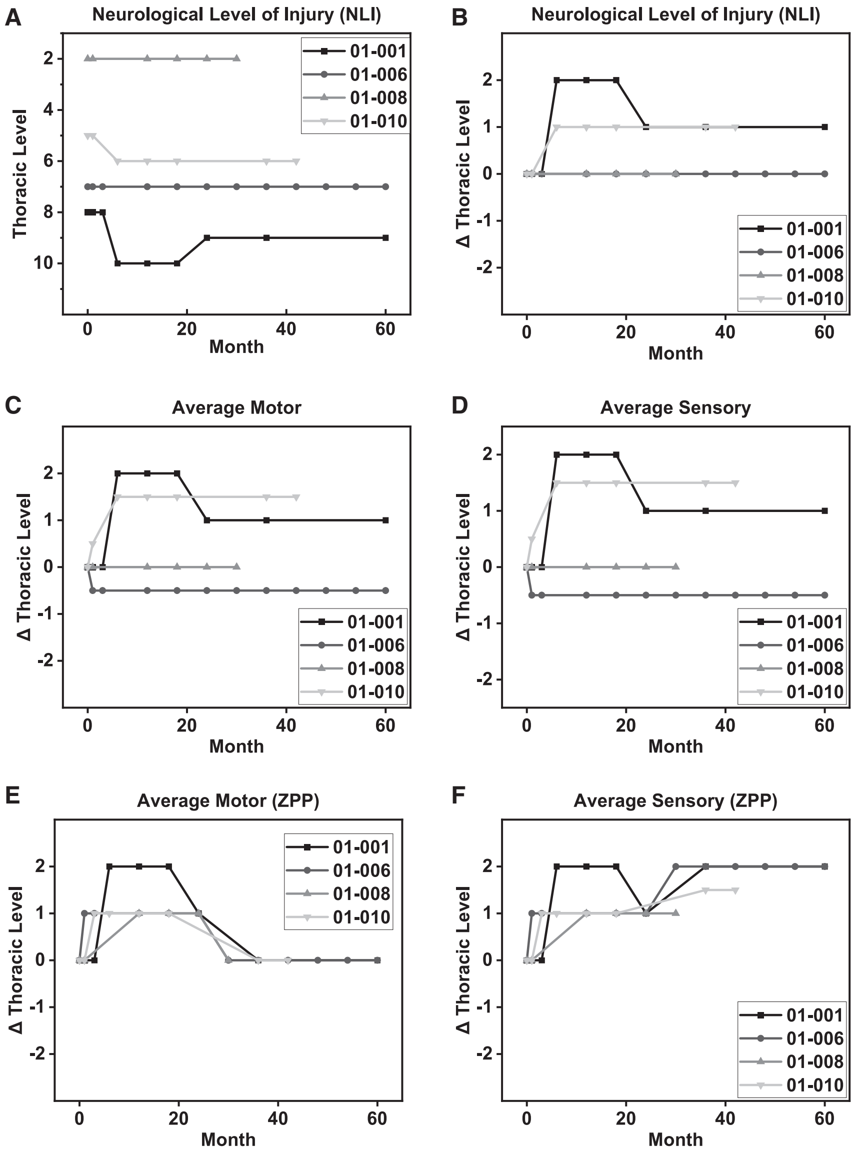

Figure 2. ISNCSCI exam scores from screening to 60 months reveals

(A and B) a one level improvement in NLI for subject 001 and 010 and remained stable for subjects 006 and 008; (C and D) average motor and sensory scores improved one level in subject 001 and 1.5 levels in subject 010; (E and F) zone of partial preservation motor remains stable across all 4 subjects, and sensory improved 1–2 levels in all 4 patients.

paraspinal muscles in three subjects. None of these outcomes translated into any functional improvements, as patients remained ASI-A complete SCI.

Electrophysiological defined improvement seen in three of four of our subjects may reflect several mechanisms including improved myelinization or development of new synaptic contacts with the host neurons and descending motor tracts via a functional reconnection of supraspinal motor centers within spinal circuitry.[7,8,26,27] Subject 001 showed two levels of sensory and motor improvement after six months post-transplant, and eventually stabilizing at one level of improvement after 27 months. Subject 010 also showed a stable improvement in one to two levels of sensory and motor levels after six months post-transplant. Similarly, subjects 001, 006, and 010 exhibited gradual two-to-four level increases in EMG activity over the course of the trial. Subject 001 (T8 level of injury) showed activity at T9–T10 at four weeks, bilateral T10 at 27 months, and T11–T12 at 60 months. Subject 006 (T7 level of injury) improved from T6–T8 at 12 weeks to T10–T11 after 42 months. Subject 010 (T5 level of injury) improved to T7 after 6 months.

The improvement in ISNCSCI scores supports the EMG findings. Alternative explanations include a significant difference in patient effort between the studies or development of spasticity, interrater variability, and multilevel innervation with paraspinal EMG. Subject 006 did not experience an improvement in ISNCSCI exam scores but did show new voluntary and EMG activity in lower muscle groups. It may be expected that subclinical reinnervation would be detected by EMG initially, prior to any manifestation of clinical improvement in ISNCSCI score.

It is important to note that sensory or motor improvement was observed within the first six months of the study. Patient 010 improved to one level of sensory and motor improvement at six months and then remained stable throughout the duration of the study. Patient 001 improved two levels of sensory and motor but dropped from two to one level of improvement at 27 months post-transplant and then remained stable (Figures 2A–2D). There were no motor or sensory improvements observed in the time after our initial preliminary publication.[5] Motor zone of partial preservation (ZPP) did observe an improvement at six months but returned to pre-study levels after 20 months post-transplantation (Figure 2E). Similarly, two of four patients showed improvement in pain measures at six months post-transplant, and any such potential improvement showed gradual decrease during the 60 months follow up (Figure 3A).

Patients with SCI are often counseled that any motor or sensory improvement usually will occur within the first two years after injury.[28] However, there are case reports of improvements after two years.[29] Thus, it is plausible that the observed post-2-year minor changes in these four patients with ASI-A SCI may be from spontaneous recovery with or without a contribution from stem cell transplantation.

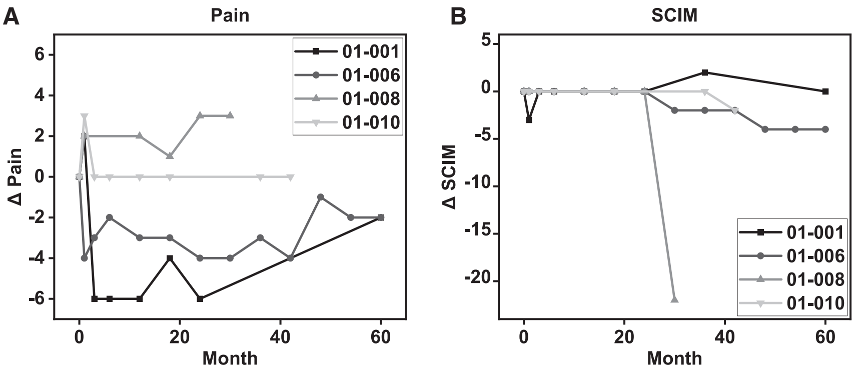

Figure 3. Pain and spinal cord independence trends from screening to month 60

(A) Change from baseline pain scores reveals improved pain scores in 2 patients, stable pain score in 1 patient, and increased pain in 1 patient.
(B) Spinal cord independence measure remained mostly stable to mildly decreased in 3 patients but significantly decreased in 1 patient.

It is also not defined whether a higher degree of synaptic formation could be achieved at higher cell doses. This safety study showed safety and tolerability and supports a further FDA-approved study in our cervical spine cohort. The dose of NSCs utilized was based on the safe and well-tolerated dose used for ALS and showed proof-of-concept results that are suggestive of functional improvement.

Limitations of the study

Overall, this trial has demonstrated encouraging secondary data, but we emphasize that the study was designed as a safety trial without statistical power, or a control group needed to fully evaluate functional changes related to NSC grafting. Nonetheless, clinical data at five years post-NSC transplantation indicate some quantifiable and tractable responses and merit further investigation with dose-escalation studies in patients with chronic SCI.

RESOURCE AVAILABILITY

Lead contact

Further information and requests for resources and reagents should be directed to and will be fulfilled by the lead contact, Joseph D. Ciacci (jciacci@ucsd.edu).

Materials availability

Requests for reagents should be directed to and will be fulfilled by the lead contact with a completed materials transfer agreement. Cell source requests should be directed to Seneca Biopharma, 20271 Goldenrod Lane Suite 2024 Germantown, MD 20876.

Data and code availability

- All data reported in this paper will be shared by the lead contact upon request.
- This paper does not report original code.
- Any additional information required to reanalyze the data reported in this paper is available from the lead contact upon request.

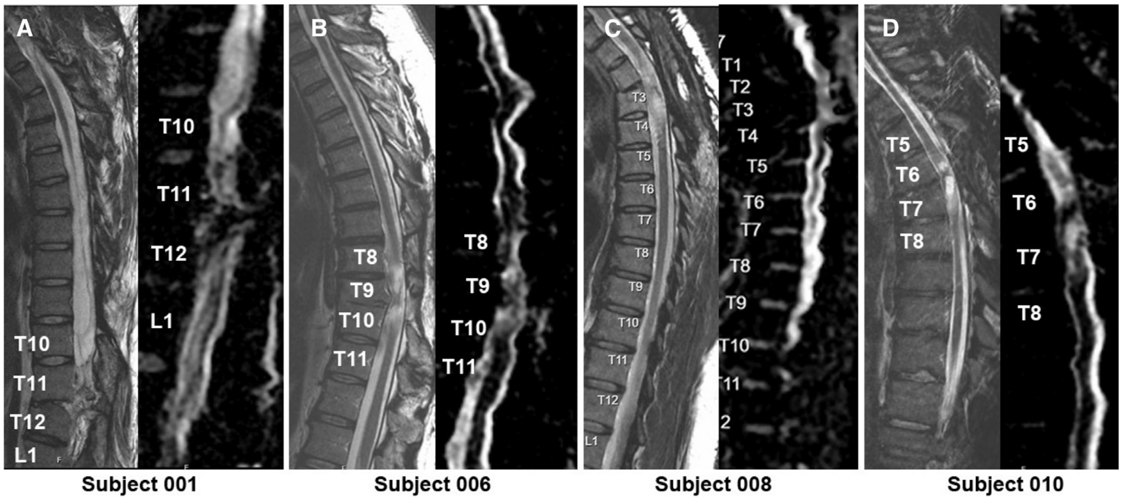

Figure 4. MRI cervical-thoracic T2 sagittal and AvDC sagittal sequences

(A) Subject 001 at 60 months (T2) and 18 months (AvDC) post-transplant (myelomalacia is seen at the area of SCI with no extension of myelomalacia or syringomyelia post-transplant), (B) subject 006 at 60 months (T2) and 30 months (AvDC) post-transplant, (C) subject 008 at 24 months (T2, AvDC) post-transplant, and (D) subject 010 at 24 months (T2, AvDC) post-transplant. In all four patients, T2 and AvDC imaging revealed a stable appearance of the spinal cord both at the injury site and rostral/caudal to the injury site.

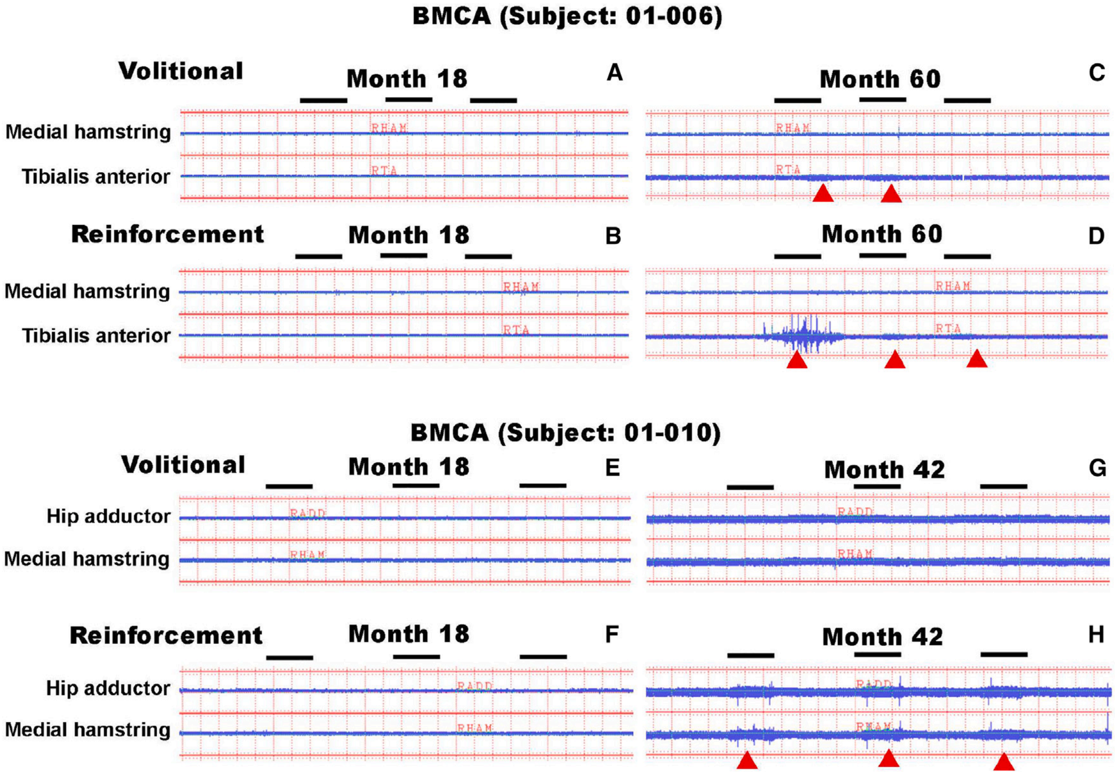

Figure 5. BMCA to identify voluntary or reinforcement maneuver-initiated EMG activity

(A and B) Recording of voluntary or reinforcement maneuver-triggered BMCA activity showed no detectable BMCA responses at 18 months after cell transplantation in subject 006.
(C and D) Subsequent recording performed at 60 months after transplantation showed suggestion of volitional EMG response in tibialis anterior (C) and after a reinforcement maneuver (D). Red arrowheads show new activity in tibialis anterior with volitional bilateral hip flexion command and reinforcement maneuver (neck flexion and deep breath). The horizontal black bar indicates onset marker.
(E and F) No detectable BMCA volitional or reinforcement maneuver response was observed at 18 months in subject 010.
(G and H) Recording at 42 months suggests new EMG activity in medial hamstring with reinforcement maneuver (H) but not with volitional command (G).

ACKNOWLEDGMENTS

This work was supported by Sanford Stem Cell Clinical Center and the CIRM UC San Diego Alpha Stem Cell Clinic.

AUTHOR CONTRIBUTIONS

From the Department of Neurosurgery, UCSD (J.R.M., D.C., M.E.A., and J.D.C.), the CIRM Alpha Stem Cell Clinic, UCSD (M. Mendoza B.C., and C.J.), and the Department of Anesthesiology (M. Marsala)—all in USA.

DECLARATION OF INTERESTS

The authors declare no competing interests.

STAR★METHODS

Detailed methods are provided in the online version of this paper and include the following:

- KEY RESOURCES TABLE
- EXPERIMENTAL MODEL AND SUBJECT DETAILS
  - Clinical trial design and patient selection
  - NSI-566 neural stem cell line
- METHOD DETAILS
  - Surgical and neural stem cell implantation procedure
  - Immunosuppression
  - Outcome measures
  - Study oversight
- QUANTIFICATION AND STATISTICAL ANALYSIS
- ADDITIONAL RESOURCES

SUPPLEMENTAL INFORMATION

Supplemental information can be found online at https://doi.org/10.1016/j.xcrm.2024.101841.

Received: June 29, 2023
Revised: June 7, 2024
Accepted: November 5, 2024
Published: December 2, 2024

REFERENCES

1. Alizadeh, A., Dyck, S.M., and Karimi-Abdolrezaee, S. (2019). Traumatic Spinal Cord Injury: An Overview of Pathophysiology, Models and Acute Injury Mechanisms. Front. Neurol. *10*, 282.
2. Badhiwala, J.H., Ahuja, C.S., and Fehlings, M.G. (2019). Time is spine: a review of translational advances in spinal cord injury. J. Neurosurg. Spine *30*, 1–18.

3. Abraham, M.E., Shalom, M., Gendreau, J., Gold, J., Pierzchajlo, G., Pierzchajlo, N., Chakravarti, S., Sahyouni, R., Murthy, N., Ciacci, J., et al. (2023). Utilizing Neuromodulation in the Treatment of Spinal Cord Injury: An Assessment of Clinical Trials from the National ClinicalTrials.gov Database. World Neurosurg. *177*, 361–367.
4. Ahuja, C.S., Mothe, A., Khazaei, M., Badhiwala, J.H., Gilbert, E.A., van der Kooy, D., Morshead, C.M., Tator, C., and Fehlings, M.G. (2020). The leading edge: Emerging neuroprotective and neuroregenerative cell-based therapies for spinal cord injury. Stem Cells Transl. Med. *9*, 1509–1530.
5. Curtis, E., Martin, J.R., Gabel, B., Sidhu, N., Rzesiewicz, T.K., Mandeville, R., Van Gorp, S., Leerink, M., Tadokoro, T., Marsala, S., et al. (2018). A First-in-Human, Phase I Study of Neural Stem Cell Transplantation for Chronic Spinal Cord Injury. Cell Stem Cell *22*, 941–950.e6.
6. Johe, K.K., Hazel, T.G., Muller, T., Dugich-Djordjevic, M.M., and McKay, R.D. (1996). Single factors direct the differentiation of stem cells from the fetal and adult central nervous system. Genes Dev. *10*, 3129–3140.
7. van Gorp, S., Leerink, M., Kakinohana, O., Platoshyn, O., Santucci, C., Galik, J., Joosten, E.A., Hruska-Plochan, M., Goldberg, D., Marsala, S., et al. (2013). Amelioration of motor/sensory dysfunction and spasticity in a rat model of acute lumbar spinal cord injury by human neural stem cell transplantation. Stem Cell Res. Ther. *4*, 57.
8. Lu, P., Wang, Y., Graham, L., McHale, K., Gao, M., Wu, D., Brock, J., Blesch, A., Rosenzweig, E.S., Havton, L.A., et al. (2012). Long-distance growth and connectivity of neural stem cells after severe spinal cord injury. Cell *150*, 1264–1273.
9. Usvald, D., Vodicka, P., Hlucilova, J., Prochazka, R., Motlik, J., Kuchorova, K., Johe, K., Marsala, S., Scadeng, M., Kakinohana, O., et al. (2010). Analysis of dosing regimen and reproducibility of intraspinal grafting of human spinal stem cells in immunosuppressed minipigs. Cell Transplant. *19*, 1103–1122.
10. Glass, J.D., Hertzberg, V.S., Boulis, N.M., Riley, J., Federici, T., Polak, M., Bordeau, J., Fournier, C., Johe, K., Hazel, T., et al. (2016). Transplantation of spinal cord-derived neural stem cells for ALS: Analysis of phase 1 and 2 trials. Neurology *87*, 392–400.
11. Levi, A.D., Anderson, K.D., Okonkwo, D.O., Park, P., Bryce, T.N., Kurpad, S.N., Aarabi, B., Hsieh, J., and Gant, K. (2019). Clinical Outcomes from a Multi-Center Study of Human Neural Stem Cell Transplantation in Chronic Cervical Spinal Cord Injury. J. Neurotrauma *36*, 891–902.
12. Levi, A.D., Okonkwo, D.O., Park, P., Jenkins, A.L., Kurpad, S.N., Parr, A.M., Ganju, A., Aarabi, B., Kim, D., Casha, S., et al. (2018). Emerging Safety of Intramedullary Transplantation of Human Neural Stem Cells in Chronic Cervical and Thoracic Spinal Cord Injury. Neurosurgery *82*, 562–575.
13. Shin, J.C., Kim, K.N., Yoo, J., Kim, I.S., Yun, S., Lee, H., Jung, K., Hwang, K., Kim, M., Lee, I.S., et al. (2015). Clinical Trial of Human Fetal Brain-Derived Neural Stem/Progenitor Cell Transplantation in Patients with Traumatic Cervical Spinal Cord Injury. Neural Plast. *2015*, 630932.
14. Tadesse, T., Gearing, M., Senitzer, D., Saxe, D., Brat, D.J., Bray, R., Gebel, H., Hill, C., Boulis, N., Riley, J., et al. (2014). Analysis of graft survival in a trial of stem cell transplant in ALS. Ann. Clin. Transl. Neurol. *1*, 900–908.
15. Lukovic, D., Moreno Manzano, V., Stojkovic, M., Bhattacharya, S.S., and Erceg, S. (2012). Concise Review: Human Pluripotent Stem Cells in the Treatment of Spinal Cord Injury. Stem Cell. *30*, 1787–1792.
16. Abraham, M.E., Martin, J., and Ciacci, J.D. (2024). Addressing challenges for repairing adult spinal cord with insights from neonates. Cell Stem Cell *31*, 585–586.
17. Regan, M.A., Teasell, R.W., Wolfe, D.L., Keast, D., Mortenson, W.B., and Aubut, J.A.L.; Spinal Cord Injury Rehabilitation Evidence Research Team (2009). A systematic review of therapeutic interventions for pressure ulcers after spinal cord injury. Arch. Phys. Med. Rehabil. *90*, 213–231.
18. Mendonca, M.V., Larocca, T.F., de Freitas Souza, B.S., Villarreal, C.F., Silva, L.F., Matos, A.C., Novaes, M.A., Bahia, C.M.P., de Oliveira Melo Martinez, A.C., Kaneto, C.M., et al. (2014). Safety and neurological assessments after autologous transplantation of bone marrow mesenchymal stem cells in subjects with chronic spinal cord injury. Stem Cell Res. Ther. *5*, 126.
19. Geffner, L.F., Santacruz, P., Izurieta, M., Flor, L., Maldonado, B., Auad, A.H., Montenegro, X., Gonzalez, R., and Silva, F. (2008). Administration of autologous bone marrow stem cells into spinal cord injury patients via multiple routes is safe and improves their quality of life: comprehensive case studies. Cell Transplant. *17*, 1277–1293.
20. Keirstead, H.S., Nistor, G., Bernal, G., Totoiu, M., Cloutier, F., Sharp, K., and Steward, O. (2005). Human embryonic stem cell-derived oligodendrocyte progenitor cell transplants remyelinate and restore locomotion after spinal cord injury. J. Neurosci. *25*, 4694–4705.
21. Cízková, D., Rosocha, J., Vanický, I., Jergová, S., and Cízek, M. (2006). Transplants of human mesenchymal stem cells improve functional recovery after spinal cord injury in the rat. Cell. Mol. Neurobiol. *26*, 1167–1180.
22. Hofstetter, C.P., Schwarz, E.J., Hess, D., Widenfalk, J., El Manira, A., Prockop, D.J., and Olson, L. (2002). Marrow stromal cells form guiding strands in the injured spinal cord and promote recovery. Proc. Natl. Acad. Sci. USA *99*, 2199–2204.
23. Nutt, S.E., Chang, E.-A., Suhr, S.T., Schlosser, L.O., Mondello, S.E., Moritz, C.T., Cibelli, J.B., and Horner, P.J. (2013). Caudalized human iPSC-derived neural progenitor cells produce neurons and glia but fail to restore function in an early chronic spinal cord injury model. Exp. Neurol. *248*, 491–503.
24. Rajasekaran, S., Kanna, R.M., and Shetty, A.P. (2012). Diffusion tensor imaging of the spinal cord and its clinical applications. J. Bone Joint Surg. Br. *94*, 1024–1031.
25. Sasiadek, M.J., Szewczyk, P., and Bladowska, J. (2012). Application of diffusion tensor imaging (DTI) in pathological changes of the spinal cord. Med. Sci. Mon. Int. Med. J. Exp. Clin. Res. : international medical journal of experimental and clinical research *18*. RA73–R79.
26. Cizkova, D., Kakinohana, O., Kucharova, K., Marsala, S., Johe, K., Hazel, T., Hefferan, M.P., and Marsala, M. (2007). Functional recovery in rats with ischemic paraplegia after spinal grafting of human spinal stem cells. Neuroscience *147*, 546–560.
27. Rosenzweig, E.S., Brock, J.H., Lu, P., Kumamaru, H., Salegio, E.A., Kadoya, K., Weber, J.L., Liang, J.J., Moseanko, R., Hawbecker, S., et al. (2018). Restorative effects of human neural stem cell grafts on the primate spinal cord. Nat. Med. *24*, 484–490.
28. McDonald, J.W., Becker, D., Sadowsky, C.L., Jane, J.A., Conturo, T.E., and Schultz, L.M. (2002). Late recovery following spinal cord injury. Case report and review of the literature. J. Neurosurg. *97*, 252–265.
29. Piepmeier, J.M., and Jenkins, N.R. (1988). Late neurological changes following traumatic spinal cord injury. J. Neurosurg. *69*, 399–402.
30. Glass, J.D., Boulis, N.M., Johe, K., Rutkove, S.B., Federici, T., Polak, M., Kelly, C., and Feldman, E.L. (2012). Lumbar intraspinal injection of neural stem cells in patients with amyotrophic lateral sclerosis: results of a phase I trial in 12 patients. Stem Cell. *30*, 1144–1151.
31. Lee, D.C., Lim, H.K., McKay, W.B., Priebe, M.M., Holmes, S.A., and Sherwood, A.M. (2004). Toward an objective interpretation of surface EMG patterns: a voluntary response index (VRI). J. Electromyogr. Kinesiol. *14*, 379–388.
32. Sherwood, A.M., McKay, W.B., and Dimitrijević, M.R. (1996). Motor control after spinal cord injury: assessment using surface EMG. Muscle Nerve *19*, 966–979.

STAR★METHODS

KEY RESOURCES TABLE

REAGENT or RESOURCE	SOURCE	IDENTIFIER
Experimental models: Cell lines		
Human fetal spinal cord-derived neural precursor line	Neuralstem Inc.	NSI-566

EXPERIMENTAL MODEL AND SUBJECT DETAILS

Clinical trial design and patient selection

This was a Phase I safety study of human spinal cord-derived neural stem cell transplantation for the treatment of chronic spinal cord injury (SCI). Chronic SCI was defined as at least one year but no more than two years after traumatic SCI. Four subjects with chronic SCI classified as AIS-A, motor and sensory complete SCI, levels T2-T12, who met eligibility criteria were enrolled. No control group was included. Inclusion and exclusion criteria are listed in our preliminary report.[5] All subjects received spinal cord injections of human spinal cord derived neural stem cells (NSI-566). The trial was registered on ClinicalTrials.gov as NCT 01772810. IRB approval was granted by UCSD Health Center, Human Research Protections Program (HRPP), 9452 Medical Center Drive, La Jolla, CA 92037.

NSI-566 neural stem cell line

NSI-566 is a human spinal cord-derived neural stem cell line that was derived from a single postmortem spinal cord of an eight-week gestational age fetus. This tissue was obtained in compliance with the National Institutes of Health (NIH) and Food and Drug Administration (FDA) Good Tissue Practice Guidelines, and under a protocol approved by an outside independent review board. Neural stem cells were isolated by dissociating a single piece of spinal cord tissue of lower cervical/upper thoracic region and expanding it as a single line.

For cell administration, NSI-566 was provided as a live-cell suspension that required no further manipulation. The cell suspension was prepared one day prior to each scheduled surgery at a cGMP facility with a final concentration of 2×10^6 cells/mL of hibernation medium. This target concentration had been established for being safe and adequate for intraspinal injections by series of preclinical[7,9] and clinical studies.[10,30] The cell suspension was then shipped to the surgery site for overnight delivery by a commercial package courier.

Before proceeding with cell administration, the cells suspension was inspected for cell viability to proceed with the implantation. The clinical lot of NSI-566 had undergone extensive preclinical safety and efficacy studies in various small and large animal studies, which had been reviewed by the US FDA under an IND (Investigational New Drug) application (#014413).

Each subject received total of six intraspinal injections (2×10^5 cells/injection delivered in 10μL of hibernation buffer). The injections were placed bilaterally into the remaining tissue lateral to the injury site and within the medial white matter-appearing tracts of approximately one segment below the injury site, as verified by intra-operative fluoroscopy imaging. Injections were made using a customized stereotactic cell injection device.[14]

METHOD DETAILS

Surgical and neural stem cell implantation procedure

The intervention included placing an anesthetized subject in the prone position and sterile processing of the associated surgical materials. An incision was performed in the posterior midline and a laminectomy was performed over the injured spinal cord segments. All prior fusion hardware was safely explanted to allow for optimum post-transplant serial magnetic resonance imaging (MRI). Following laminectomy, a small incision was made in the dura allowing exposure of the injured spinal cord segment. The stereotaxic injection frame platform was then attached to percutaneous posts above and below the laminectomy site.[14] The injection device consisted of a Z-drive holding a beveled needle in perpendicular position over the exposed spinal cord. The top end of the needle was attached to tubing which was attached to a microprocessor-controlled syringe pump. The syringe was backfilled with mineral oil to eliminate air and to create an immiscible barrier against aqueous solution in the syringe. The syringe plunger was inserted into the syringe and attached to the drive spindle of the injection pump. Separately, the injection cannula was manually filled with sterile injectable saline to eliminate air and loaded with the cell suspension. Bilateral injection positions were determined by preoperative MRI and targeted approximately 1 mm lateral to tissue bordering the injury site. The needle was lowered into the spinal cord to the depth of approximately 4 mm from the pial surface. The guide sheath was retracted which converted the cannula into a “floating cannula.” This feature allowed for accuracy of delivery without suspension of respiration. The cell suspension was then injected using the syringe pump at flow rate of 5.0 μL/min for a period of 2 min. The needle was left in place for 1 min after injection and then slowly pulled out of the spinal cord for all six injections. After all injections were completed, the dura was then closed in a watertight fashion, and the

posterior spinal fascia and skin was closed in meticulous layers. Subjects were then extubated, followed by recovery in post-anesthesia care unit and intermediate level care unit of the hospital.

Immunosuppression

All four subjects were initiated and maintained for 12 weeks on a combination cocktail of immunosuppressive (IS) regimen[14,30]: Basiliximab (Simulect) 20 mg intravenous (IV) administered within 2 h prior to transplantation surgery and second dose of 20 mg on post-transplant day three or four. Tacrolimus was started on post-transplant day one (0.1 mg/kg/day every 12 h by mouth, trough level 4–8 ng/mL). Mycophenolate mofetil was started on post-transplant day one at 500 mg twice a day, increased to 500 mg in the morning and 1 gm at night on post-transplant day eight, and increased to 1 gm twice per day on post-transplant day 15. Tacrolimus and mycophenolate mofetil were then weaned after 12 weeks post-transplantation. Medications were reduced by half at weeks 13 and 14, followed by complete cessation at week 15. The presence of antibodies against donor HLAs were monitored. Changes in MRI intensity at the cell transplant area were also monitored before and after the IS withdrawal.

Outcome measures

Primary outcome measures included adverse events and clinically significant laboratory abnormalities. Additional secondary outcome assessments were made to measure any post-operative changes. Quality of life scores and physical exams were conducted, including ISNCSCI (International Standards for the Neurological Classification of Spinal Cord Injury), SCIM (Spinal Cord Independence Measure), Functional Independence Measure (FIM), allodynia and neuropathic pain, and bowel and bladder follow-up. Neurophysiological changes were monitored when feasible by needle electromyography (EMG) and/or surface poly-electromyography Brain Motor Control Assessment (BMCA).[31,32] Graft survival in the transplant site was determined by MRI and via autopsy if one is completed. Imaging studies were performed using standard 1.5–3.0T MRI for safety monitoring. Diffusion tensor imaging in this study used both 1.5T GE Signa HDxt and 3.0T GE Discover 750w MRI scanners, single B0 scan, with 15 directions at b = 600 s/mm2. 2D diffusion weighted-EPI used flip angle 90, TR 2500-5000ms, TE 64-95ms, matrix size 128 × 32–38x8-12, FOV 200–340 × 200–340 × 32–48 mm3, 3.5-4mm slice thickness, 8–12 slices, pixel BW = 1953 Hz/pixel, PE direction = left to right, and 1 measurement. The effectiveness of immunosuppression was determined by absence of donor-specific HLA antibodies. Subjects were followed postoperatively at two weeks, monthly for six months, and at every six months for up for total 60 months post stem cell treatment. Patients did not receive any additional rehabilitation beyond their routine outpatient physical and occupational therapy.

Study oversight

An independent Data Safety Monitoring Board (DSMB) was convened at approximately four-week intervals to review the available safety data. The DSMB was tasked with making specific recommendations regarding study continuation. It did not identify any safety issues which precluded continuation of the study.

QUANTIFICATION AND STATISTICAL ANALYSIS

The data collected from these methods were analyzed descriptively, given the small sample size. No control group was included, and statistical power calculations were not applicable. The outcomes were primarily presented in terms of individual observed values rather than inferential statistical tests.

ADDITIONAL RESOURCES

The trial was registered in ClinicalTrials.gov as NCT01772810 and supported by the UC San Diego Sanford Stem Cell Clinical Center and the CIRM UC San Diego Alpha Stem Cell Clinic.

Cell Reports Medicine, Volume *5*

Supplemental information

Long-term clinical and safety outcomes from a single-site phase 1 study of neural stem cell transplantation for chronic thoracic spinal cord injury

Joel R. Martin, Daniel Cleary, Mickey E. Abraham, Michelle Mendoza, Betty Cabrera, Catriona Jamieson, Martin Marsala, and Joseph D. Ciacci

SUPPLEMENTARY MATERIAL

Table S1: Supplementary electrophysiology summary data. Related to Table 2, Figure 5.

<table>
<tr><td>Subject 001</td><td>

Week 4
some activity T6-10 RA and PS bilaterally
none in right Rectus femoris
Week 6
some activity T7-9 RA and PS bilaterally
none in right Rectus femoris
Month 6
some activity T7-9 RA and PS bilaterally
none in right Rectus femoris
Month 12
some activity T7-9 RA and PS bilaterally
none in right Rectus femoris
Month 18
only BMCA done.
Month 27

PSL:
Multifidus: T9 with many MUPs; T10 with none
Superficial: T10 with many MUPs; T11 with none/+spont
PSR:
Multifidus: T10 with several MUPs; T11 with none/+spont
Superficial: T10 with several MUPs; T11 with none/+spont
RAL: Upper segment with many MUPs
RAR: Upper segment with several MUPs
LL bilaterally: no voluntary MUPs; +spont LVm, RTA, RVm
BMCA: newly developed muscle responsiveness in lower limbs to reinforcement maneuvers

Month 36
No EMG changes.
Month 60
EMG: Needle EMG now indicates some voluntary control of rectus abdominis bilaterally at T11 and T12 at the left, not seen previously. No MUPs under voluntary control at T11 on the right, as before.
BMCA: Constant EMG activity was recorded from the right tibialis anterior muscle and constant, low amplitude movement of the right great toe was seen. This seemed to be involuntary (spontaneous).

</td></tr>
<tr><td>Subject 006</td><td>

Week 12
PSL or MTF: no MUPs T7 or below
PSR or MTF: MUPs noted at T6-8
RAR: no MUPs at T7 (presumably lower-mid segment)
Month 12

PSL
Superficial: MUPs noted at T6-8
Multifidus: T6 with many MUPs; T7 with none
PSR
Superficial: MUPs noted at T6-8
Multifidus: T6 with many MUPs; T7 with none
RAL: no MUPs
RAR: 2 MUPs distant in upper segment

Month 18

PSR: new intolerance to study at T9 and above
PSL: new intolerance to study at T9 and above
T9 and 10 with 1-2 superficial MUPs (not tested before)
RA: new intolerance to study at T9 and above
no definite MUPs at T9-T10

Month 36
Continued intolerance to study at T9 and above. Examined T12,T11,T10 bilaterally
RAR

</td></tr>
</table>

<table>
<tr><td></td><td>

Right rectus abdominus at T10- +2 spont activity, +2 complex repetitive discharges, 1 voluntary MUP at T11- no spont activity/discharges, no MUPs at T12- +1 spont activity, no complex repetitive discharges, 2 voluntary MUPs

RAL

At T10 +1spont activity, +2complex repetitive discharges, no voluntary MUPs
At T11- no spont activity/discharges, no MUPs
At T12- no spont activity/discharges, 1 MUP (possibly 2) under voluntary control

Control of rectus abdominus muscles bilat at T10 and to a lesser extent at T12 ; At T10, level of activation seemed better on left;
EMG findings were not present in the T10-T12 paraspinal muscles

Month 42

EMG: right sural sensory amplitude is larger than last study, but still significantly smaller than left. Right tibial motor CMAP lower limb EMG is unchanged. Rectus abdominus at and below umbilicus (T10-T12) shows no voluntary MUPs. MEPs still do not show continuity of central motor pathways to lower trunk and lower limbs.

Month 48

EMG: Sural sensory potential is now present and quite normal (suggesting technical problem during last study), right rectus abdominis did not show any MUPs. rectus abdominus at T11 with 1 MUP and T12 with 2-3 MUPs; Paraspinal muscles showed MUPs under voluntary control at T10-T11 bilaterally, but not at T12.
BMCA: no change

Month 54

EMG No change
BMCA: No change from baseline, touch of the patient introduced 60 Hz interference

Month 60

EMG: left superficial peroneal sensory response is absent now. Rectus abdominis EMG did not show MUPs on either side. T11-T12 Paraspinals not studied due to T8-T10 showing no MUPs during visit. Lower Limb EMG showed no voluntary MUPs
BMCA: suggestion of response of right tibialis anterior to reinforcement maneuver

</td></tr>
<tr><td>Subject 008</td><td>

Month 12

RAR: no activation, unclear sampling

Month 18

RA: no activation bilaterally
PSR: need to confirm level of landmark (8cm above base of scar)
PSL: need to confirm level of landmark (13cm above base of scar)

</td></tr>
<tr><td>Subject 010</td><td>

Week 12

RAL: 2-3 hard to activate MUPs likely in superior segment
RAR: no MUPs
PSL (Not tested above T9 due to scar)

Multifidus: None at T9 or below
Superficial: None at T9 or below

PSR

Multifidus: T6 with many MUPs, T7 with none, +spont
Superficial: not reported as different to Multifidus.

Month 6

RAL: No MUPs (likely lower segment tested)
RAR: not tested
PSL: Voluntary MUPs in left T5-T8 (likely superficial)
PSR: 1 MUP in T7 (unclear if superficial or Multifidus); T8 with none

Month 18

LL bilaterally: No MUPs, mild spontaneous activity in right TA
PSL

Multifidus: T6 with chronic neurogenic changes; T7 with none
Superficial: T8 with several units; T9 with none

PSR

Multifidus: T6 with 2-3 voluntary units; T7 none
Superficial: T7 with 1-2 MUPs; T8 with none

</td></tr>
</table>

	◦ RAL: 2-3 hard to activate MUPs likely in superior segment<br>◦ RAR: no MUPs<br>• Month 36<br>◦ EMG: SPSR reconfirmed at T7<br>◦ BMCA: Deep tendon reflexes increased at knees<br>• Month 42<br>◦ EMG: No motor units under voluntary control in lower limbs<br>◦ BMCA: Deep tendon reflexes increased at knees, suggestion of response of right medial hamstring to reinforcement maneuver

EMG electromyography, BMCA brain motor control assessment, PS paraspinal, PSL paraspinal left, PSR paraspinal right, SPSR superficial paraspinal right, RA rectus abdominis, RAL rectus abdominis left, RAR rectus abdominis right, LL lower limbs, LVm left vastus medius, RTA right tibialis anterior, RVm right vastus medius, MTF multifidus, MUP multi-unit potential, CMAP, MEP motor evoked potential.
